# Net contribution and predictive ability of the CUN-BAE body fatness index in relation to cardiometabolic conditions

**DOI:** 10.1007/s00394-018-1743-9

**Published:** 2018-06-11

**Authors:** Veronica Davila-Batista, Antonio J. Molina, Laura Vilorio-Marqués, Leila Lujan-Barroso, Fernanda de Souza-Teixeira, Rocío Olmedo-Requena, Jorge Arias de la Torre, Lidia García-Martínez, Laura Álvarez-Álvarez, Heinz Freisling, Javier Llorca, Miguel Delgado-Rodríguez, Vicente Martin

**Affiliations:** 1 The Research Group in Gene-Environment and Health Interactions (GIIGAS), University of León, León, Spain; 2 Instituto de Biomedicina (IBIOMED), University of León, León, Spain; 3 CIBER of Epidemiology and Public Health (CIBERESP), Madrid, Spain; 4 Unit of Nutrition and Cancer, Cancer Epidemiology Research Program, Catalan Institute of Oncology-IDIBELL, L’Hospitalet de Llobregat, Barcelona, Spain; 5 Department of Nursing of Public Health, Mental Health and Maternity and Child Health, School of Nursing, Universitat de Barcelona, L’Hospitalet de Llobregat, Barcelona, Spain; 6 Exercise and Neuromuscular System Research Group, Superior School of Physical Education, Federal University of Pelotas, Pelotas, Brazil; 7 Department of Preventive Medicine and Public Health, Instituto de Investigación Biosanitaria de Granada (Ibs.Granada), Complejo Hospitalario Universitario de Granada, University of Granada, Granada, Spain; 8 Agency for Health Quality and Assessment of Catalonia (AQuAS), Barcelona, Spain; 9 Section of Nutrition and Metabolism, International Agency for Research on Cancer (IARC-WHO), Lyon, France; 10 Universidad de Cantabria, IDIVAL, Santander, Spain; 11 Universidad de Jaén, Jaén, Spain; 12 Área de Medicina Preventiva y Salud Pública, Facultad de Ciencias de la Salud, Universidad de León, 24071 León, Spain

**Keywords:** Body fatness, CUN-BAE, BMI, Hypertension, Diabetes, Metabolic syndrome

## Abstract

**Background:**

The CUN-BAE (Clínica Universidad de Navarra-Body adiposity estimator) index is an anthropometric index based on age, sex and body mass index (BMI) for a refined prediction of body fatness in adults. CUN-BAE may help detect metabolically unhealthy individuals with otherwise normal weight according to BMI or waist circumference (WC). The aim of this study was to evaluate whether CUN-BAE, independent of its components (BMI, age and sex), was associated with cardiometabolic conditions including arterial hypertension, diabetes mellitus and metabolic syndrome (MetS).

**Methods:**

The ENRICA study was based on a cross-sectional sample of non-institutionalized men and women representative of the adult Spanish population. Body weight, height, and WC were measured in all participants. The residual of CUN-BAE (rCUN-BAE), i.e. the part of the index not explained by its components, was calculated. The associations of CUN-BAE, rCUN-BAE, BMI and WC with hypertension, diabetes and MetS were analysed by multivariate logistic regression, and the Akaike information criterion (AIC) was calculated.

**Results:**

The sample included 12,122 individuals. rCUN-BAE was associated with hypertension (OR 1.14, 95% CI 1.07–1.21) and MetS (OR 1.48, 1.37–1.60), but not with diabetes (OR 1.05, 0.94–1.16). In subjects with a BMI < 25 kg/m^2^, CUN-BAE was significantly associated with all three outcome variables. CUN-BAE was more strongly associated with the cardiometabolic conditions than BMI and WC and fit similar AICs.

**Conclusions:**

The CUN-BAE index for body fatness was positively associated with hypertension, diabetes and MetS in adults independent of BMI or WC. CUN-BAE may help to identify individuals with cardiometabolic conditions beyond BMI, but this needs to be confirmed in prospective settings.

**Electronic supplementary material:**

The online version of this article (10.1007/s00394-018-1743-9) contains supplementary material, which is available to authorized users.

## Background

Excess adiposity is estimated to cause 4 million deaths and approximately 4.9% of years of life lost worldwide, and the relationships with cardiometabolic conditions including metabolic syndrome, type 2 diabetes, and cardiovascular diseases are well established [1]. Moreover, the prevalence of obesity is continuing to increase worldwide [2, 3].

In the general population, the most widely used measures to define excess body fatness (BF) are body mass index (BMI) and waist circumference (WC). These anthropometric indicators have been criticized because they do not take into account important factors involved with adiposity, in particular age, sex, or race [3–5]. The use of more accurate methods to assess body fatness in large epidemiological studies, such as dual-energy X-ray absorptiometry (DXA) or air-displacement plethysmography (Bod-Pod) is hampered by high costs. Therefore, there is great interest in accurate and cost-efficient indicators of BF that can predict disease risk better than BMI or WC.

A recently developed alternative anthropometric method that deserves special consideration is the CUN-BAE (Clínica Universidad de Navarra-Body Adiposity Estimator) index. This estimator of percentage of body fat is based on BMI, sex and age of Caucasian subjects [6]. Previous CUN-BAE assessments showed a stronger correlation with cardiovascular risk factors related to adiposity than BMI or WC [7–9]. The CUN-BAE index may help detect individuals who are of normal weight according to BMI, but are metabolically unhealthy [10, 11]. In this regard, the CUN-BAE index has already been used as an anthropometric measure to try to reach a consensus on the definitions of metabolic health [12].

However, since CUN-BAE is based on BMI, sex and age, its relationship with cardiometabolic conditions may be due to the components of this indicator. Knowing whether a composite index, such as the CUN-BAE index, adds a significant predictive ability of cardiometabolic risk apart from its components is relevant.

The aim of this study was to evaluate the independent and net contribution of the body fatness estimator, CUN-BAE, in the prediction of cardiometabolic conditions in Spanish adults.

## Materials and methods

### Study design

This report is based on the Study on Nutrition and Cardiovascular Risk in Spain (ENRICA study) [13], a cross-sectional study performed between 2008 and 2010 with a national representative sample of 12,948 non-institutionalized adults in Spain. The participants were selected by multistage cluster sampling. First, the sample was stratified by province and municipality size. Then, clusters were randomized by municipalities and census section. Finally, households were selected by random telephone dialling in each section.

For this study, we selected Caucasian participants with complete available anthropometric information (6.4% participants in the ENRICA study were excluded).

### Ethical considerations

All participants signed a written informed consent. The ENRICA study protocol was approved by the Clinical Research Ethics Committees of the University Hospital La Paz in Madrid and Hospital Clínic in Barcelona.

### Study variables

Data on anthropometry, blood pressure and biological samples were collected during two home visits. Further, a telephone interview with a structured questionnaire on lifestyle and other variables of interest was carried out [13].

### Anthropometric measurement

Body weight, height and WC were measured twice in each subject by trained staff according to standard conditions [13, 14] using electronic scales (model Seca 841, precision to 0.1 kg) and portable extendable stadiometers (model Ka We 44,444 Seca). BMI was calculated as weight in kilograms divided by squared height in metres: kg/m^2^. The classification of BMI was defined by the World Health Organization [2]: normal weight < 25 kg/m^2^, overweight ≥ 25 kg/m^2^ and obesity ≥ 30 kg/m^2^. WC was measured in centimetres using flexible, inelastic belt-type tapes. The standard CUN-BAE was calculated using the equation suggested by Gómez-Ambrosi et al. [7]: %BF = − 44.988 + (0.503 × age) + (10.689 × sex) + (3.172 × BMI) − (0.026 × BMI2) + (0.181 × BMI × sex) − (0.02 × BMI × age) − (0.005 × BMI2 × sex) + (0.00021 × BMI2 × age), where age is measured in years and sex was codified as 0 for men and 1 for women.

### Outcomes

Blood pressure was measured with validated automatic sphygmomanometers (model Omron M6) according to standardized procedures. In summary, two sets of blood pressure readings (with three measured) were separated by 90 min. Arterial hypertension (ATH) was defined as systolic blood pressure ≥ 140 mm Hg, diastolic blood pressure ≥ 90 mm Hg, and/or current treatment with an antihypertensive drug [15].

Biochemical markers in blood and urine were obtained after a 12-h fasting period [16]. Diabetes mellitus (DM) was defined following recommendations of the American Diabetes Association [16, 17] as a 12-h fasting serum glucose ≥ 126 mg/dl, glycosylated haemoglobin (HbA1c) ≥ 6.5%, or treatment with oral antidiabetic drugs or insulin [16, 17].

Serum high-density lipoprotein cholesterol was measured by elimination/catalase using the direct method, and triglycerides were measured using the glycerol phosphate oxidase method (ADVIA de Siemens).

Metabolic syndrome (MetS) includes physiological indicators such as systolic blood pressure ≥ 130 mmHg, diastolic ≥ 85 mmHg or treatment with antihypertensive medication, impaired glucose (a fasting blood glucose level ≥ 100 mg/dL or treatment with antidiabetic drugs), elevated triglycerides (≥ 150 mg/dL), low serum levels of high-density lipoprotein (HDL < 40 mg/dL in men or < 50 mg/dL in women) and abdominal obesity (WC ≥ 102 cm in men and ≥ 88 cm in women). The diagnosis of MetS was based on the presence of at least three of these five criteria, using the International Diabetes Federation consensus [18].

### Other variables

The following variables were considered as potential confounders: age, sex, education level (categories were based on the Spanish education system: primary school, secondary school, and higher education), civil status (single, married, and separated/divorced/widower), smoking (current, past, and never) and alcohol intake (never drinkers, former drinkers, moderate drinkers, and heavy drinkers).

### Statistical analysis

Descriptive analyses of sociodemographic and lifestyle characteristics, anthropometric data and cardiometabolic disease by sex were carried out using common procedures.

### Estimation of the residual CUN-BAE (rCUN-BAE)

The rCUN-BAE was estimated through the residual method [19, 20]. In a linear model, we regressed the BMI and age as independent variables and the CUN-BAE index as the dependent variable. We then computed the rCUN-BAE: difference between observed CUN-BAE figures and those predicted by the model; by definition, residuals have zero correlation (are orthogonal) with the observed CUN-BAE. The residuals were calculated separately for men and women. Thus, the rCUN-BAE may contribute to the body fat estimator developed by Gomez-Ambrosi independently of the BMI and age elements.

Additionally, residual CUN-BAE values were estimated using WC and age (rCUN-BAE2) to ensure that associations between the percentage of total body fat and outcomes were independent of abdominal body fat.

#### Statistical model

We used multivariate logistic regression models to estimate associations of anthropometric measurements (CUN-BAE, BMI and WC) with AHT, DM and MetS. All analyses were a priori stratified by sex. The exposure variables were analysed both on the original scale and per 1-SD increment and, in supplementary analyses, as categorical variables using quartiles. Linear trend tests were performed.

Three different multivariate logistic models were fit separately for each of the three cardiometabolic conditions. For these, a crude model was fit separately for each of the three exposure measures, and multivariable models were adjusted for the potential confounders including level of education, civil status, alcohol consumption, smoking status and age for BMI and WC, but without age for CUN-BAE, in which age is already included in the calculation (base Model 1). Additionally, CUN-BAE was analysed within categories of BMI cut-offs.

In a second model, associations of rCUN-BAE together with BMI and age were introduced in combination with confounding variables of base Model 1. Furthermore, in a third model, the rCUN-BAE2, WC and age were considered as independent variables.

Results are reported as multivariable-adjusted odds ratios (ORs) with 95% confidence intervals (CIs). All reported p values are two-tailed with a statistical significance of *p* < 0.05.

Additionally, the Akaike information criterion (AIC) was calculated to determine which model best fits the data.

All analyses were carried out with the Stata/SE 14 software package (College Station, Texas, USA).

## Results

General and anthropometric characteristics of the study population by sex are described in Table 1. The study population consisted of 12,122 adult Spanish Caucasian participants, 5749 men (47.4%) and 6373 women. The mean age was 47.4 years (range: 18–96 years; SD 16.67). In this population 32.4% had ATH, 6.6% had DM and 23.1% had MetS. The BMI and WC mean values for men were higher than those among women. Inversely, CUN-BAE, indicative of adiposity, showed a lower mean of 27.4% fat in men compared to 37.4% in women.

**Table 1:** Baseline characteristics of the study population

	Men		Women	
	*n* (5749)	%	*n* (6373)	%
Education level				
Primary school	1399	24.4	2113	33.3
Secondary school	2614	45.6	2460	38.7
University	1715	29.9	1779	28.0
Civil status				
Single	1504	26.5	1538	24.5
Married	3910	68.9	3829	61.1
Separated/divorced/widower	258	4.6	904	14.4
Smoking				
Current	1670	29.2	1597	25.1
Past	1842	32.2	1222	19.2
Never	2216	38.7	3533	55.6
Alcohol intake				
Never drinkers	1183	21.0	2991	47.9
Ex drinkers	297	5.3	328	5.3
Moderate drinkers	3686	65.3	2689	43.1
Heavy drinkers	480	8.5	234	3.8
Arterial hypertension	2243	39.3	1652	26.2
Diabetes	461	8.1	329	5.2
Metabolic syndrome	1561	27.7	1183	19.0
	**Mean**	**SD**	**Mean**	**SD**
Age	47.2	16.46	47.7	16.86
CUN-BAE (BF%)	27.4	6.23	37.4	7.32
BMI (kg/m^2^)	27.5	4.05	26.2	5.08
Waist circumference (cm)	96.8	11.86	85.6	13.38
rCUN-BAE for BMI + age	0.0	0.75	0.0	1.23
rCUN-BAE2 for WC + age	0.0	2.81	0.0	3.36

*BMI* body mass index (km/m^2^)

*CUN-BAE* Clínica Universidad de Navarra-Body Adiposity Estimator calculated using the Gómez-Ambrosi equation (body fat %)

*WC* waist circumference (cm)

*rCUN-BAE and rCUN-BAE2*: residual CUN-BAE and residual CUN-BAE2. Residuals were calculated with separate sex-specific linear regression models with age and BMI or WC as the independent variables and CUN-BAE as the dependent variable

Pearson’s correlations between anthropometric measures, except for residuals, were moderate-to-strong and varied according to anthropometric indicator and sex (Supplementary Table S1).

**Fig. 1:**
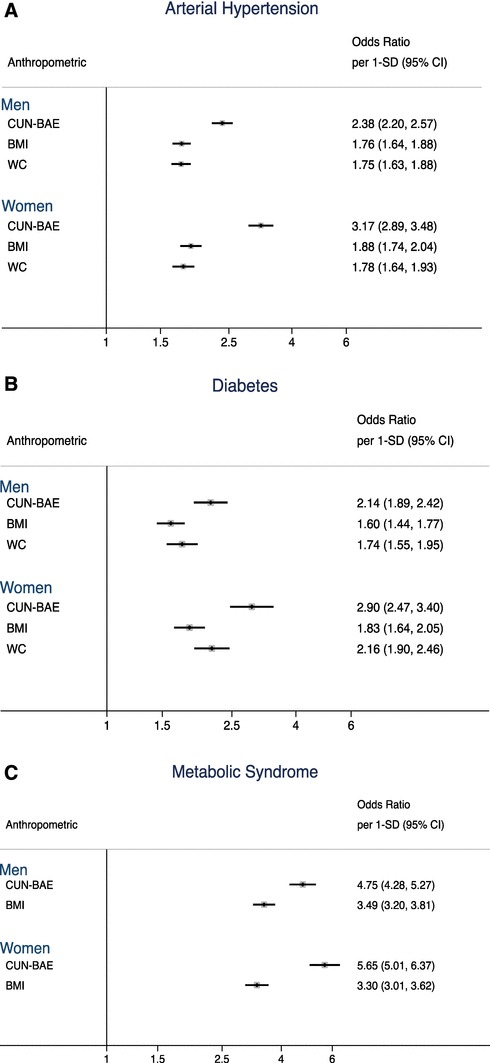
**Odds ratio for the association per 1-SD increment of standard anthropometric measure with cardiometabolic conditions, by sex.a** Odds ratio for the association of anthropometric measures with arterial hypertension. **b** Odds ratio for the association of anthropometric measures with diabetes. **c** Odds ratio for the association of anthropometric measures with metabolic syndrome. Odds ratio (Model 1) was adjusted for age, education level, civil status, alcohol drinker and current smoker; age not into the CUN-BAE analyses were not adjusted for age because age was already included in the CUN-BAE. *1-SD* One standard deviation, *BMI* body mass index (km/m^2^), *CUN-BAE* Clínica Universidad de Navarra-Body Adiposity Estimator (body fat %), *WC* waist circumference (cm)

Figure 1 shows the separate associations between anthropometric measures and cardiometabolic conditions (base Model 1). Standard CUN-BAE, BMI and WC were significantly positively associated with AHT, DM and MetS. The ORs per 1-SD increment of the cardiometabolic conditions were indicative of a strong association with CUN-BAE than with the other anthropometric measures. Statistically significant differences were observed by sex. The ORs per 1-SD increment of CUN-BAE were indicative of an association with ATH (OR 1-SD = 2.38, 95% CI 2.20–2.57 in men; OR 3.17, 95% CI 2.89–3.48 in women), DM (OR 2.14, 95% CI 1.89–2.42 in men; OR 2.90, 95% CI 2.47–3.40 in women) and MetS (OR 4.75, 95% CI 4.28–5.27 in men; OR 5.65, 95% CI 5.01–6.37 in women).

Results for the original scale and categorical analyses are shown in Supplementary Table S2. The highest categories of CUN-BAE, BMI and WC (q1 vs q4) were significantly associated with an increase in cardiometabolic conditions, showing similar associations by sex. All categories showed a significant linear trend.

Table 2 shows the associations between rCUN-BAE and the outcomes. The rCUN-BAE, after being mutuality adjusted for BMI and age (Model 2), was significantly associated with AHT (OR 1.14, 95% CI 1.07–1.21) and MetS (OR 1.48, 95% CI 1.37–1.60). In contrast, rCUN-BAE was not associated with DM. Supplementary Table 3 shows the results for rCUN-BAE2 accounting for WC and age (Model 3). The OR for rCUN-BAE2 was also indicative of significant association with AHT and MetS. In addition, rCUN-BAE2 was associated with DM.

**Table 2:** Odds ratio for the association of anthropometric measures, rCUN-BAE and BMI mutually adjusted, with cardiometabolic conditions using the residual method

Model 2	All						Men						Women					
	OR*	95% CI		OR**	95% CI		OR*	95% CI		OR**	95% CI		OR*	95% CI		OR**	95% CI	
Arterial hypertension																		
rCUN-BAE	**1.14**	1.07	1.21	**1.14**	1.07	1.21	**1.17**	1.05	1.30	**1.18**	1.06	1.32	**1.18**	1.09	1.28	**1.15**	1.06	1.26
BMI	**1.15**	1.14	1.16	**1.15**	1.14	1.16	**1.15**	1.13	1.17	**1.16**	1.14	1.18	**1.15**	1.13	1.17	**1.14**	1.13	1.17
Age	**1.08**	1.07	1.08	**1.08**	1.07	1.08	**1.06**	1.06	1.06	**1.06**	1.05	1.07	**1.10**	1.09	1.11	**1.10**	1.10	1.11
Sex W	**0.46**	0.41	0.50	**0.43**	0.39	0.48												
Diabetes																		
rCUN-BAE	1.05	0.95	1.16	1.05	0.94	1.16	0.98	0.82	1.18	0.98	0.81	1.17	1.11	0.98	1.27	1.10	0.96	1.26
BMI	**1.14**	1.11	1.17	**1.14**	1.11	1.17	**1.12**	1.08	1.16	**1.12**	1.08	1.16	**1.16**	1.12	1.21	**1.15**	1.11	1.20
Age	**1.07**	1.07	1.08	**1.07**	1.06	1.08	**1.07**	1.06	1.08	**1.06**	1.05	1.07	**1.08**	1.07	1.09	**1.07**	1.06	1.09
Sex W	**0.55**	0.47	0.64	**0.48**	0.40	0.59												
Metabolic syndrome																		
rCUN-BAE	**1.47**	1.36	1.59	**1.48**	1.37	1.60	**1.46**	1.26	1.70	**1.47**	1.26	1.72	**1.55**	1.41	1.70	**1.56**	1.42	1.73
BMI	**1.35**	1.33	1.37	**1.35**	1.33	1.38	**1.39**	1.36	1.43	**1.40**	1.36	1.43	**1.34**	1.31	1.37	**1.34**	1.30	1.37
Age	**1.05**	1.04	1.05	**1.05**	1.04	1.05	**1.04**	1.03	1.04	**1.04**	1.03	1.04	**1.07**	1.06	1.07	**1.07**	1.06	1.08
Sex W	**0.56**	0.50	0.62	**0.51**	0.45	0.57												

ORs* (residual method) were adjusted for rCUN-BAE, BMI and age (continuous variables) and sex only in the model combining men and women (in all: 0 men, 1 woman)

ORs** (residual method) were adjusted for rCUN-BAE, BMI and age plus sex, educational level, civil status, alcohol drinker and current smoker

*BMI* body mass index (kg/m^2^), *rCUN-BAE* residual CUN-BAE. Residuals were calculated with separate sex-specific linear regression models with age and BMI as the independent variable and CUN-BAE as the dependent variables

The bold values significance that 95% CI are statistically significant

A slightly better predictive fit according to AIC was obtained for models using rCUN-BAE2 (Table 3).

**Table 3:** Post-estimation Akaike information criterion (AIC)

	Men	Women
Arterial hypertension		
CUN-BAE	6295.19	5208.05
BMI	6025.89	4575.70
WC	6061.02	4633.89
rCUN-BAE (for BMI + age)	6019.16	4577.29
rCUN-BAE2 (for WC + age)	6013.32	4575.11
Diabetes		
CUN-BAE	2736.49	1993.09
BMI	2592.34	1902.53
WC	2579.89	1879.17
rCUN-BAE (for BMI + age)	2594.27	1911.56
rCUN-BAE2 (for WC + age)	2579.62	1877.92
Metabolic syndrome		
CUN-BAE	4879.86	4036.39
BMI	4900.97	4029.09
rCUN-BAE (for BMI + age)	4878.84	3955.19
rCUN-BAE2 (for WC + age)	4644.10	3806.55

All models were adjusted for age, education level, civil status, alcohol drinker and current smoker; the standard CUN-BAE analyses were not adjusted for age because age was already increased in the CUN-BAE. Estimate of the odds ratio for the associated of anthropometric measures with cardiometabolic conditions, in agreement with continuous variable models

*AIC* Akaike information criterion, *BMI* body mass index (km/m^2^), *CUN-BAE* Clinica Universidad de Navarra-Body Adiposity Estimator (body fat %), *WC* waist circumference (cm), *rCUN-BAE* CUN-BAE residual were calculated with age and BMI as the independent variable and CUN-BAE as the dependent variables, *rCUN-BAE2* CUN-BAE residual were calculated with age and WC as the independent variable and CUN-BAE as the dependent variables

Table 4 shows the results of the CUN-BAE analyses within categories of BMI. In subjects with a normal BMI (< 25 kg/m^2^), CUN-BAE was strongly associated with cardiometabolic conditions, with an OR per 1-SD increment of AHT of 6.73 (95% CI 5.29–8.55), OR per 1-SD increment of DM of 4.10 (95% CI 2.43–6.94) and OR per 1-SD increment of MetS of 7.36 (95% CI 4.86–11.16). Additionally, in overweight and obesity BMI categories, CUN-BAE was also significantly positively associated with cardiometabolic conditions (Table 4).

**Table 4:** Odds ratio for the association of CUN-BAE with cardiometabolic conditions, within BMI categories

CUN-BAE	Categories of BMI								
	< 25 kg/m^2^			25–30 kg/m^2^			≥ 30 kg/m^2^		
	OR	95% CI		OR	95% CI		OR	95% CI	
Arterial hypertension									
All									
Continuous	**1.33**	1.29	1.38	**1.38**	1.34	1.43	**1.14**	1.10	1.17
1-SD	**6.73**	5.29	8.55	**8.14**	6.67	9.93	**2.30**	1.90	2.79
Men									
Continuous	**1.26**	1.19	1.33	**1.33**	1.28	1.38	**1.10**	1.06	1.14
1-SD	**4.19**	3.03	5.80	**5.84**	4.63	7.37	**1.80**	1.41	2.31
Women									
Continuous	**1.41**	1.33	1.48	**1.49**	1.41	1.57	**1.17**	1.12	1.22
1-SD	**12.09**	8.29	17.63	**18.36**	12.37	27.24	**3.09**	2.27	4.21
Diabetes mellitus									
Al									
Continuous	**1.24**	1.14	1.34	**1.38**	1.30	1.46	**1.16**	1.12	1.20
1-SD	**4.10**	2.43	6.94	**8.03**	5.44	11.85	**2.74**	2.17	3.45
Men									
Continuous	**1.24**	1.12	1.39	**1.37**	1.27	1.47	**1.17**	1.11	1.22
1-SD	**3.89**	1.98	7.67	**7.05**	4.48	11.11	**2.61**	1.93	3.53
Women									
Continuous	**1.23**	1.09	1.39	**1.38**	1.24	1.54	**1.16**	1.10	1.22
1-SD	**4.52**	1.86	10.96	**10.84**	4.91	23.93	**2.97**	2.04	4.30
Metabolic Syndrome									
All									
Continuous	**1.35**	1.27	1.44	**1.38**	1.33	1.43	**1.18**	1.15	1.22
1-SD	**7.36**	4.86	11.16	**7.90**	6.29	9.92	**3.04**	2.49	3.72
Men									
Continuous	**1.23**	1.13	1.35	**1.33**	1.28	1.39	**1.17**	1.12	1.23
1-SD	**3.71**	2.14	6.43	**6.02**	4.59	7.89	**2.70**	2.07	3.54
Women									
Continuous	**1.46**	1.33	1.60	**1.46**	1.37	1.55	**1.19**	1.14	1.24
1-SD	**16.06**	8.24	31.29	**15.73**	10.18	24.30	**3.55**	2.61	4.83

*OR* odds ratio (standard multivariable method, Model 1) was adjusted for education level, civil status, alcohol drinker, current smoker and sex

*1-SD* one standard deviation increment, *BMI* body mass index (km/m^2^), *%BF* percentage of body fat, *CUN-BAE* Clinica Universidad de Navarra-Body Adiposity Estimator, Gómez-Ambrosi equation (body fat %)

## Discussion

In this representative cross-sectional sample of Spanish adults, we found that CUN-BAE was significantly positively associated with cardiovascular conditions independent of its components (BMI, age and sex). To date, this is the first study to evaluate the net effects of the body fatness estimator CUN-BAE.

The CUN-BAE equation takes into account age, sex and BMI [7]. These classic variables are well established to be consistent cardiometabolic risk factors. In the current study, the residual rCUN-BAE values, together adjusted for BMI, age and sex, were associated with AHT and MetS. Therefore, constant values of the CUN-BAE equation add information, independent of its design components. The rCUN-BAE provided similar estimates in men and women. Both the BMI and rCUN-BAE models had similar adjusted estimates using AIC. However, rCUN-BAE did not have a statistically significant relationship with DM. Most likely, the sum of the elements age, sex and BMI explains the association between CUN-BAE and DM. However, rCUN-BAE2 was associated with all cardiometabolic conditions examined, including a positive association with DM. Thus, the AIC model values were similar, but a little higher in the adjusted models using rCUN-BAE2 than in those using rCUN-BAE, BMI and WC. Other authors observed that CUN-BAE had a strong correlation with metabolic markers such as glucose, HOMA and leptin [6, 9, 21, 22]. Therefore, rCUN-BAE may capture the consequences of excess adiposity, at least AHT and MetS, that are not justified by BMI, sex and age.

In this study, the CUN-BAE equation by Gomez-Ambrosi [6] showed a stronger significate association with the cardiometabolic conditions examined than BMI or WC. Similarly, previous studies have shown that CUN-BAE could be a useful index for cardiovascular conditions [6, 8, 9, 12]. In this line with this, CUN-BAE has been shown to better correlate with BF% than BMI [7, 22, 23]. Furthermore, we should highlight that body fat is an active endocrine organ that affects the inflammatory state and circulating hormone levels, such as leptin, as well as insulin resistance and levels of triglycerides, cholesterol and oestrogens. Excess adiposity stimulates the inflammatory response of the body, potentially contributing to the creation of an environment that encourages changes associated with these metabolic conditions [3, 24].

The CUN-BAE equation was developed according to the Bod-Pod method to design a better predictor for body fatness. The author’s objective in designing the CUN-BAE equation was to develop a better predictor of metabolic health factors and identify a phenotype of metabolically unhealthy individuals [7]. The individuals who have a normal BMI, but are metabolically unhealthy could be an interesting risk group to identify. In this study, among the individuals identified as non-overweight or non-obese according to BMI cut-off points, increasing CUN-BAE was significantly associated with all cardiometabolic conditions, and this association was stronger in women than in men. In the NHANES study using DXA, CUN-BAE was the best predictor of BF% among the BMI-built equations. Therefore, CUN-BAE has come to be of special consideration as an estimate of BF in non-Hispanic whites and in females [23].

One of the strengths of this study is the representativeness of the sample of the adult population of Spain. In addition, anthropometric data and blood pressure were acquired for all study participants. In this regard, the ENRICA study has the highest response rate (51%) as evaluated by the European Health Examination Survey [25].

Methodologically, we used the residual method to assess the additional contribution of a composite index apart from its components [19, 26–28]. The estimation of residuals is commonly used to study nutrients adjusted by total caloric intake [19, 29], and now this method is beginning to be more commonly used to examine abdominal vs overall obesity [20, 29–31]. However, the ENRICA study is a cross-sectional study, and the results should be interpreted cautiously due to limitations related to the establishment of the direction of the causal relationship between variables. Further, there could be bias due to confounding variables associated with cardiometabolic condition such as physical activity levels or their diet. Such a bias could be inflated by the high proportion of overweight or obesity in this study if these individuals differed significantly in their activity or dietary behaviour from the source population. However, other national surveys also show that more than half of the adult Spanish population are overweight or obese. Another possible limitation of this study is that the sample included only Caucasian people; therefore, these results may not be applicable to other ethnicities. An inherent limitation of the body fatness estimator CUN-BAE is that the equation was developed using a sample acquired for other purposes, and that most of the individuals included in the study performed a low level of physical activity. Nevertheless, CUN-BAE presented a high correlation with BF measured by DXA in other studies.

## Conclusions

The CUN-BAE index for body fatness was associated with cardiometabolic conditions in adults independent of BMI or WC. CUN-BAE may help to identify individuals with cardiometabolic conditions beyond BMI, but this needs to be confirmed in prospective settings.

## Electronic supplementary material

Below is the link to the electronic supplementary material.

Supplementary material 1 (DOCX 48 KB)

## Abbreviations

AHT: Arterial hypertension
BF: Body fat
BMI: Body mass index
CUN-BAE: Clínica Universidad de Navarra-Body adiposity estimator, Gómez-Ambrosi equation
DM: Diabetes mellitus
MetS: Metabolic syndrome
rCUN-BAE: CUN-BAE residual was calculated with age and BMI as the independent variable and CUN-BAE as the dependent variable
rCUN-BAE2: CUN-BAE residual was calculated with age and WC as the independent variable and CUN-BAE as the dependent variable
WC: Waist circumference
