# Supplementary material for: Net contribution and predictive ability of the CUN-BAE body fatness index in relation to cardiometabolic conditions
Source: Eur J Nutr. 2018 Jun 11;58(5):1853–61. doi: 10.1007/s00394-018-1743-9 (PMC6647072; doi:10.1007/s00394-018-1743-9)
Supplement: Supplementary file 1 — Supplementary material 1 (DOCX 48 KB) [file 394_2018_1743_MOESM1_ESM.docx]

**Net contribution and predictive ability of the CUN-BAE body fatness index in relation to cardiometabolic conditions.**

**Supporting information**

**S1 Table.** Pearson correlation of anthropometric measures.

**S2 Table.** Odds ratio for the association of standard anthropometric measures with cardiometabolic conditions.

**S3 Table.** Odds ratio for the association of anthropometric measures, rCUN-BAE2 and WC mutually adjusted, with cardiometabolic conditions using the residual method.

**Supplementary Table S1.** Pearson correlation of anthropometric measures.

|  | CUN-BAE | BMI | WC | rCUN-BAE | rCUN-BAE2 | Age |
| --- | --- | --- | --- | --- | --- | --- |
| **All** |  |  |  |  |  |  |
| CUN-BAE | 1 |  |  |  |  |  |
| BMI | 0.67 | 1 |  |  |  |  |
| WC | 0.39 | 0.82 | 1 |  |  |  |
| rCUN-BAE (for BMI + age) | 0.60 | -0.13 | -0.39 | 1 |  |  |
| rCUN-BAE2 (for WC + age) | 0.69 | 0.15 | -0.34 | 0.86 | 1 |  |
| Age | 0.49 | 0.33 | 0.40 | 0.02 | 0.01 | 1 |
| **Men** |  |  |  |  |  |  |
| CUN-BAE | 1 |  |  |  |  |  |
| BMI | 0.94 | 1 |  |  |  |  |
| WC | 0.87 | 0.85 | 1 |  |  |  |
| rCUN-BAE (for BMI + age) | 0.12 | 0.00 | 0.03 | 1 |  |  |
| rCUN-BAE2 (for WC + age) | 0.45 | 0.50 | 0.00 | 0.22 | 1 |  |
| Age | 0.55 | 0.26 | 0.42 | 0.00 | 0.00 | 1 |
| **Women** |  |  |  |  |  |  |
| CUN-BAE | 1 |  |  |  |  |  |
| BMI | 0.94 | 1 |  |  |  |  |
| WC | 0.84 | 0.84 | 1 |  |  |  |
| rCUN-BAE (for BMI + age) | 0.17 | 0.00 | 0.05 | 1 |  |  |
| rCUN-BAE2 (for WC + age) | 0.46 | 0.50 | 0.00 | 0.28 | 1 |  |
| Age | 0.64 | 0.38 | 0.46 | 0.00 | 0.00 | 1 |

BMI: Body mass index (km/m^2^); CUN-BAE: Clínica Universidad de Navarra - Body Adiposity Estimator (body fat %); WC: Waist circumference (cm); rCUN-BAE: residual CUN-BAE. Residuals were calculated with separate sex-specific linear regression models with age and BMI or WC as the independent variable and CUN-BAE as the dependent variables.

**Supplementary Table S2.** Odds ratio for the association of standard anthropometric measures with cardiometabolic conditions.

| **Men** | | | | | | | | **Women** | | | | | | |
| --- | --- | --- | --- | --- | --- | --- | --- | --- | --- | --- | --- | --- | --- | --- |
| **Measure** | **N** | **n** | **Prev** | **OR** | **95% CI** | | **Measure** | | **N** | **n** | **Prev** | **OR** | **95% CI** | |
| **Arterial hypertension** | | | | | | | | | | | | | | |
| **CUNBAE** |  |  |  |  |  |  |  | |  |  |  |  |  |  |
| *continuous* |  |  |  | ***1.15*** | *1.14* | *1.16* | *continuous* | |  |  |  | ***1.17*** | *1.16* | *1.19* |
| q1 (≤ 23.31) | 1422 | 157 | 11.0 | 1 (ref) |  |  | q1 (≤ 31.96) | | 1578 | 39 | 2.5 | 1 (ref) |  |  |
| q2 (23.32 - 27.77) | 1429 | 435 | 30.4 | **2.80** | 2.26 | 3.46 | q2 (31.97 - 37.39) | | 1585 | 193 | 12.2 | **4.04** | 2.82 | 5.78 |
| q3 (27.78 - 31.68) | 1429 | 741 | 51.9 | **6.26** | 5.06 | 7.73 | q3 (37.40 - 42.59) | | 1582 | 528 | 33.4 | **11.21** | 7.92 | 15.85 |
| q4 (≥ 31.69) | 1426 | 910 | 63.8 | **9.81** | 7.91 | 12.17 | q4 (≥ 42.60) | | 1571 | 892 | 56.8 | **25.00** | 17.65 | 35.42 |
| p trend ⌃ |  |  |  | <0.001 |  |  | p trend ⌃ | |  |  |  | <0.001 |  |  |
| **BMI** |  |  |  |  |  |  |  | |  |  |  |  |  |  |
| *continuous* |  |  |  | ***1.15*** | *1.13* | *1.17* | *continuous* | |  |  |  | ***1.13*** | *1.12* | *1.15* |
| q1 (≤ 24.70) | 1424 | 255 | 17.9 | 1 (ref) |  |  | q1 (≤ 22.56) | | 1578 | 115 | 7.3 | 1 (ref) |  |  |
| q2 (24.71- 27.11) | 1425 | 478 | 33.5 | **1.77** | 1.46 | 2.15 | q2 (22.57 - 25.34) | | 1583 | 255 | 16.1 | 1.27 | 0.97 | 1.66 |
| q3 (27.12 - 29.86) | 1432 | 675 | 47.1 | **2.74** | 2.26 | 3.31 | q3 (25.35 - 29.03) | | 1587 | 483 | 30.4 | **2.04** | 1.58 | 2.64 |
| q4 (≥ 29.87) | 1425 | 835 | 58.6 | **4.45** | 3.67 | 5.40 | q4 (≥ 29.04) | | 1568 | 799 | 51.0 | **4.42** | 3.42 | 5.71 |
| p trend ⌃ |  |  |  | <0.001 |  |  | p trend ⌃ | |  |  |  | <0.001 |  |  |
| **Waist circumference** | |  |  |  |  |  |  | |  |  |  |  |  |  |
| *continuous* |  |  |  | ***1.05*** | *1.04* | *1.05* | *continuous* | |  |  |  | ***1.04*** | *1.04* | *1.05* |
| q1 (≤ 88.70) | 1427 | 203 | 14.2 | 1 (ref) |  |  | q1 (≤ 75.25) | | 1583 | 90 | 5.7 | 1 (ref) |  |  |
| q2 (88.71 - 96.60) | 1427 | 490 | 34.3 | **2.07** | 1.70 | 2.54 | q2 (75.26 - 84.25) | | 1586 | 263 | 16.6 | **1.59** | 1.20 | 2.10 |
| q3 (96.61 - 104.45) | 1432 | 689 | 48.1 | **3.12** | 2.55 | 3.81 | q3 (84.26 - 94.05) | | 1587 | 503 | 31.7 | **2.31** | 1.76 | 3.02 |
| q4 (≥ 104.46) | 1420 | 861 | 60.6 | **4.73** | 3.86 | 5.80 | q4 (≥ 94.06) | | 1560 | 796 | 51.0 | **4.54** | 3.47 | 5.93 |
| p trend ⌃ |  |  |  | <0.001 |  |  | p trend ⌃ | |  |  |  | <0.001 |  |  |
| **Diabetes** | | | | | | | | | | | | | | |
| **CUNBAE** |  |  |  |  |  |  |  | |  |  |  |  |  |  |
| *continuous* |  |  |  | ***1.13*** | *1.11* | *1.15* | *continuous* | |  |  |  | ***1.16*** | *1.13* | *1.18* |
| q1 (≤ 23.31) | 1412 | 23 | 1.6 | 1 (ref) |  |  | q1 (≤ 31.96) | | 1572 | 9 | 0.6 | 1 (ref) |  |  |
| q2 (23.32 - 27.77) | 1410 | 62 | 4.4 | **2.07** | 1.25 | 3.41 | q2 (31.97 - 37.39) | | 1574 | 19 | 1.2 | **1.27** | 0.55 | 2.92 |
| q3 (27.78 - 31.68) | 1421 | 137 | 9.6 | **3.90** | 2.43 | 6.26 | q3 (37.40 - 42.59) | | 1566 | 66 | 4.2 | **3.82** | 1.84 | 7.92 |
| q4 (≥ 31.69) | 1420 | 239 | 16.8 | **7.18** | 4.52 | 11.41 | q4 (≥ 42.60) | | 1563 | 235 | 15.0 | **11.92** | 5.86 | 24.22 |
| p trend ⌃ |  |  |  | <0.001 |  |  | p trend ⌃ | |  |  |  | <0.001 |  |  |
| **BMI** |  |  |  |  |  |  |  | |  |  |  |  |  |  |
| *continuous* |  |  |  | ***1.12*** | *1.09* | *1.15* | *continuous* | |  |  |  | ***1.12*** | *1.09* | *1.15* |
| q1 (≤ 24.70) | 1411 | 50 | 3.5 | 1 (ref) |  |  | q1 (≤ 22.56) | | 1574 | 16 | 1.0 | 1 (ref) |  |  |
| q2 (24.71- 27.11) | 1411 | 88 | 6.2 | 1.30 | 0.89 | 1.89 | q2 (22.57 - 25.34) | | 1568 | 31 | 2.0 | 1.28 | 0.65 | 2.50 |
| q3 (27.12 - 29.86) | 1423 | 119 | 8.4 | **1.45** | 1.01 | 2.08 | q3 (25.35 - 29.03) | | 1570 | 79 | 5.0 | **2.25** | 1.22 | 4.16 |
| q4 (≥ 29.87) | 1418 | 204 | 14.4 | **2.81** | 1.99 | 3.95 | q4 (≥ 29.04) | | 1563 | 203 | 13.0 | **4.88** | 2.70 | 8.82 |
| p trend ⌃ |  |  |  | <0.001 |  |  | p trend ⌃ | |  |  |  | <0.001 |  |  |
| **Waist circumference** | |  |  |  |  |  |  | |  |  |  |  |  |  |
| *continuous* |  |  |  | ***1.05*** | *1.04* | *1.06* | *continuous* | |  |  |  | ***1.06*** | *1.05* | *1.07* |
| q1 (≤ 88.70) | 1422 | 30 | 2.1 | 1 (ref) |  |  | q1 (≤ 75.25) | | 1578 | 10 | 0.6 | 1 (ref) |  |  |
| q2 (88.71 - 96.60) | 1407 | 73 | 5.2 | **2.07** | 1.70 | 2.54 | q2 (75.26 - 84.25) | | 1575 | 30 | 1.9 | **1.52** | 0.70 | 3.28 |
| q3 (96.61 - 104.45) | 1417 | 125 | 8.8 | **3.12** | 2.55 | 3.81 | q3 (84.26 - 94.05) | | 1570 | 70 | 4.5 | **2.42** | 1.17 | 4.97 |
| q4 (≥ 104.46) | 1417 | 233 | 16.4 | **4.73** | 3.86 | 5.80 | q4 (≥ 94.06) | | 1552 | 219 | 14.1 | **6.63** | 3.30 | 13.32 |
| p trend ⌃ |  |  |  | <0.001 |  |  | p trend ⌃ | |  |  |  | <0.001 |  |  |
| **Metabolic Syndrome** | | | | | | | | | | | | | | |
| **CUNBAE** |  |  |  |  |  |  |  | |  |  |  |  |  |  |
| *continuous* |  |  |  | ***1.28*** | *1.26* | *1.31* | *continuous* | |  |  |  | ***1.27*** | *1.25* | *1.29* |
| q1 (≤ 23.31) | 1402 | 49 | 3.5 | 1 (ref) |  |  | q1 (≤ 31.96) | | 1562 | 11 | 0.7 | 1 (ref) |  |  |
| q2 (23.32 - 27.77) | 1408 | 203 | 14.4 | **4.56** | 3.25 | 6.40 | q2 (31.97 - 37.39) | | 1570 | 61 | 3.9 | **5.03** | 2.55 | 9.93 |
| q3 (27.78 - 31.68) | 1420 | 428 | 30.1 | **11.49** | 8.26 | 15.98 | q3 (37.40 - 42.59) | | 1561 | 306 | 19.6 | **28.26** | 14.86 | 53.74 |
| q4 (≥ 31.69) | 1413 | 881 | 62.4 | **44.28** | 31.81 | 61.62 | q4 (≥ 42.60) | | 1546 | 805 | 52.1 | **115.09** | 60.62 | 218.52 |
| p trend ⌃ |  |  |  | <0.001 |  |  | p trend ⌃ | |  |  |  | <0.001 |  |  |
| **BMI** |  |  |  |  |  |  |  | |  |  |  |  |  |  |
| *continuous* |  |  |  | ***1.36*** | *1.33* | *1.39* | *continuous* | |  |  |  | ***1.27*** | *1.24* | *1.29* |
| q1 (≤ 24.70) | 1404 | 72 | 5.1 | 1 (ref) |  |  | q1 (≤ 22.56) | | 1562 | 23 | 1.5 | 1 (ref) |  |  |
| q2 (24.71- 27.11) | 1405 | 204 | 14.5 | **2.73** | 2.04 | 3.65 | q2 (22.57 - 25.34) | | 1564 | 88 | 5.6 | **2.97** | 1.82 | 4.84 |
| q3 (27.12 - 29.86) | 1423 | 426 | 29.9 | **6.29** | 4.77 | 8.29 | q3 (25.35 - 29.03) | | 1569 | 317 | 20.2 | **9.89** | 6.26 | 15.63 |
| q4 (≥ 29.87) | 1411 | 859 | 60.9 | **23.80** | 18.10 | 31.29 | q4 (≥ 29.04) | | 1544 | 755 | 48.9 | **33.82** | 21.50 | 53.18 |
| p trend ⌃ |  |  |  | <0.001 |  |  | p trend ⌃ | |  |  |  | <0.001 |  |  |

ORs (base Model 1) were adjusted for age, studies performed, civil status, alcohol drinker and current smoker; age not into the CUN-BAE analyses were not adjusted for age because age was already included in the CUN-BAE.

BMI: Body mass index (km/m^2^); CUN-BAE: Clínica Universidad de Navarra - Body Adiposity Estimator (body fat %); WC: Waist circumference (cm); ⌃ ptrend: p tendence lineal value; prev: prevalence.

**Table 3.** Odds ratio for the association of anthropometric measures, rCUN-BAE and WC mutually adjusted, with cardiometabolic conditions using the residual method.

| **Model 3** | All | | | | | |  | Men | | | | | |  | Women | | | | | |
| --- | --- | --- | --- | --- | --- | --- | --- | --- | --- | --- | --- | --- | --- | --- | --- | --- | --- | --- | --- | --- |
|  | OR* | 95% CI | | OR** | 95% CI | |  | OR* | 95% CI | | OR** | 95% CI | |  | OR* | 95% CI | | OR** | 95% CI | |
| **Arterial Hypertension** | | | | | | | | | | | | | | | | | | | | |
| rCUN-BAE2 | **1.09** | 1.08 | 1.11 | **1.09** | 1.07 | 1.11 |  | **1.09** | 1.07 | 1.11 | **1.09** | 1.06 | 1.11 |  | **1.11** | 1.08 | 1.13 | **1.11** | 1.08 | 1.13 |
| WC | **1.05** | 1.04 | 1.05 | **1.05** | 1.04 | 1.05 |  | **1.05** | 1.04 | 1.05 | **1.05** | 1.04 | 1.06 |  | **1.05** | 1.04 | 1.05 | **1.05** | 1.04 | 1.05 |
| Age | **1.07** | 1.07 | 1.08 | **1.07** | 1.07 | 1.08 |  | **1.05** | 1.05 | 1.06 | **1.05** | 1.05 | 1.06 |  | **1.10** | 1.09 | 1.10 | **1.10** | 1.09 | 1.11 |
| Sex | **0.67** | 0.61 | 0.75 | **0.63** | 0.56 | 0.70 |  |  |  |  |  |  |  |  |  |  |  |  |  |  |
| **Diabetes** | | | | | | | | | | | | | | | | | | | | |
| rCUN-BAE2 | **1.04** | 1.01 | 1.06 | **1.03** | 1.00 | 1.06 |  | **1.03** | 0.99 | 1.07 | 1.03 | 0.99 | 1.07 |  | **1.05** | 1.01 | 1.09 | **1.04** | 1.00 | 1.08 |
| WC | **1.06** | 1.05 | 1.06 | **1.06** | 1.05 | 1.06 |  | **1.05** | 1.04 | 1.06 | **1.05** | 1.04 | 1.06 |  | **1.06** | 1.05 | 1.07 | **1.06** | 1.05 | 1.07 |
| Age | **1.07** | 1.06 | 1.07 | **1.06** | 1.05 | 1.07 |  | **1.06** | 1.05 | 1.07 | **1.06** | 1.05 | 1.07 |  | **1.07** | 1.06 | 1.08 | **1.07** | 1.05 | 1.08 |
| Sex | 0.92 | 0.78 | 1.08 | **0.77** | 0.63 | 0.94 |  |  |  |  |  |  |  |  |  |  |  |  |  |  |
| **Metabolic Syndrome** | | | | | | | | | | | | | | | | | | | | |
| rCUN-BAE2 | **1.10** | 1.08 | 1.12 | **1.09** | 1.07 | 1.11 |  | **1.09** | 1.06 | 1.12 | **1.09** | 1.06 | 1.12 |  | **1.11** | 1.09 | 1.14 | **1.11** | 1.08 | 1.13 |
| WC | **1.13** | 1.12 | 1.13 | **1.13** | 1.12 | 1.13 |  | **1.14** | 1.13 | 1.15 | **1.14** | 1.13 | 1.15 |  | **1.12** | 1.11 | 1.13 | **1.12** | 1.11 | 1.13 |
| Age | **1.03** | 1.03 | 1.03 | **1.03** | 1.02 | 1.03 |  | **1.02** | 1.01 | 1.02 | **1.02** | 1.01 | 1.02 |  | **1.05** | 1.04 | 1.05 | **1.05** | 1.04 | 1.06 |
| Sex | **1.66** | 1.48 | 1.85 | **1.45** | 1.28 | 1.65 |  |  |  |  |  |  |  |  |  |  |  |  |  |  |

ORs* (residual method) were adjusted for rCUN-BAE, waist circumference and age (continuous variables) and sex only in the model combining men and women (in all: 0 men, 1 women).

ORs** (residual method) were adjusted for rCUN-BAE, waist circumference and age plus sex, educational level, civil status, alcohol drinker and current smoker.

Abbreviations:

CUN-BAE: Clínica Universidad de Navarra - Body Adiposity Estimator (body fat %); WC: Waist circumference (cm); rCUNBAE2: residual CUN-BAE2. Residuals were calculated with separate sex-specific linear regression models with age and WC as the independent variable and CUN-BAE as the dependent variables.
